# Behavioral Health Services Outcomes That Matter Most to Caregivers of Children, Youth, and Young Adults with Mental Health Needs

**DOI:** 10.3390/ijerph21020172

**Published:** 2024-02-01

**Authors:** Genevieve Graaf, Katherine Kitchens, Millie Sweeney, Kathleen C. Thomas

**Affiliations:** 1 School of Social Work, University of Texas at Arlington, Arlington, TX 76019, USA; 2 Family-Run Executive Director Leadership Association (FREDLA), Elliot City, MD 21042, USA; 3 Division of Pharmaceutical Outcomes and Policy, Eshelman School of Pharmacy, University of North Carolina, Chapel Hill, NC 27599, USA; 4 Cecil G. Sheps Center for Health Services Research, University of North Carolina, Chapel Hill, NC 27599, USA

**Keywords:** youth mental health, child mental health, parent support in child mental health, caregiver perspectives

## Abstract

This project documents the service outcomes that caregivers value most. A diverse group of caregivers, representing six regions of the United States, participated in two rounds of virtual one-hour focus groups. In round 1, participants identified what they hoped to gain from using behavioral health services for themselves, their families, and their child and discussed what made services a positive experience for them. They then reported their top-three most-hoped-for outcomes. In round 2, groups validated and refined summary findings from round 1. Caregivers prioritized service quality outcomes, primarily. They expressed a desire for an accessible, respectful, and supportive treatment environment, underpinned by well-trained and culturally responsive professionals. Caregivers also desire seamless cross-sector provider collaboration and care transitions, which integrate the insights and preferences of families and children themselves to craft a customized care plan. Priority outcomes not related to service quality included hoping to gain increased knowledge, resources, and tools and techniques to support the mental health needs of their children, to see their children improve their daily functioning and for their child develop more effective interpersonal communication skills. Caregivers also reported hoping to experience less stigma related to the mental health needs of their children and to achieve personal fulfillment for themselves and their children. Research, policies, and mental health services should prioritize and be designed to address the outcomes that matter to youth and families.

## 1. Introduction

Federal policies increasingly advocate for centering the perspectives and values of youth, families, and caregivers in child and adolescent behavioral health care [1] as health systems seek to become more patient-centered [2]. Developing services through policy and research that deliver what families value may increase family engagement in services and reduce attrition, helping families achieve their goals more quickly [3]. Considering the outcomes that matter most to caregivers may be especially pivotal in increasing service engagement because caregivers play a central role in their family’s participation in mental health treatment [4]. Caregivers provide legal consent for their child to receive care, arrange and schedule treatment visits and interactions, often provide transportation related to treatment, and must participate in service planning, assessment, and some interventions [5,6]. 

Despite the many reasons that incorporating the outcome priorities of youth and caregiver service users might be important, the value and preferences of service users are notably underrepresented in the development of mental health service outcome frameworks and the ongoing debates about the merits of various outcomes [7]. In particular, caregiver perspectives and priorities are often absent in mental health services research and are largely overlooked in healthcare system reform efforts [8]. A small body of existing research has attempted to understand service user priorities. It suggests that caregivers are concerned with reducing their child’s symptoms, improving coping skills, social skills, family and peer relationships, school, vocational and independent functioning, and behavior management [9,10,11,12,13,14,15,16]. However, these studies on how caregivers perceive and define service effectiveness are limited by narrow sampling frames (e.g., families currently using services or families of youth with specific diagnoses), secondary data sources (e.g., use of administrative records regarding treatment goals or target problems), or geographic limitations (e.g., studies are limited to one community or behavioral health clinic). Further, these studies often fail to examine the values and reasons behind caregiver preferences, which could provide valuable, actionable insights into the needs of caregivers and their children. As such, there remains a significant knowledge gap concerning the outcomes that families utilizing mental health services prioritize and why these outcomes matter to them. 

This lack of comprehensive data underscores the necessity to broaden our understanding of service effectiveness from the viewpoint of those directly impacted by these services. To address this need, this study draws on community-based samples of youth and young adults (YYA) and caregivers from six communities across the United States to broadly understand what outcomes matter most to the YYAs and caregivers who engage in child and youth mental health services. A secondary aim of this study was to uncover the values and beliefs underlying caregiver outcome priorities. While the overarching study examined these aims for both YYAs and caregivers, the complexity and breadth of the findings necessitated separate reports. We present the results pertinent to caregivers in this paper. We detail the findings related to YYAs in a separate paper [17].

## 2. Methods

### 2.1. Data Sources and Sample

We conducted this collaborative study to investigate caregiver-prioritized outcomes in behavioral health services in partnership with the Family-Run Executive Director Leadership Association (FREDLA). FREDLA represents a robust national network of family-run organizations (FROs), staffed mainly by caregivers with lived experience caring for children and youth with behavioral health needs. These FROs, which span local, regional, and state levels, aim to provide targeted services and support to families of children, youth, and young adults with behavioral challenges. They frequently coordinate with other behavioral health agencies to ensure a cohesive service delivery model. Collectively, these organizations reach an extensive constituency, offering training and support to over 100,000 families each year.

FREDLA, in collaboration with the research team, sourced the sample for our study from its network of over 100 local and statewide FROs. We utilized a stratified sampling technique to ensure we gathered a diverse and representative cross-section of caregivers, which was instrumental in capturing a wide array of experiences and perspectives on the valued outcomes in behavioral health services. We actively selected and compensated six FROs to form local partnerships and assist with recruiting participants. Selection criteria for these FROs included geographic, clinical, and racial and ethnic diversity. In selecting partner FROs, we sought representation from each U.S. Census region and prioritized those with higher levels of racial and ethnic diversity in the families they served. Leveraging FREDLA’s intimate knowledge of its FRO network, we also prioritized FROs with capacity to engage in research activities. We selected organizations representing families from North Carolina (South Atlantic), Arizona (West Mountain), Nevada (West Mountain), Washington (West Pacific), Pennsylvania (Northeast), and Mississippi (South Central), ensuring our sample encompassed diverse regional characteristics from urban to rural and frontier environments. This strategy was crucial in obtaining a sample that genuinely reflects the complex nature of caregiver experiences nationwide.

We partnered with local FROs to identify potential participants. Each FRO recruited six to eight caregivers who exhibited interest and availability to participate, aiming for approximately six participants per focus group. FROs invited parents or caregivers of children, youth, or young adults of any age. FROs were asked to recruit participants representing a range of racial, ethnic, educational, and economic backgrounds, as well as participants with a wide range of experience with behavioral health services. These organizations engaged with potential participants individually, extending invitations to participate in the project and providing them with a comprehensive project summary and other recruitment materials. The research team, FREDLA, and the partnering FROs jointly developed these materials. We invited participants to attend two one-hour focus groups conducted via the video conferencing platform Zoom, spaced approximately two months apart. FROs offered respondents a monetary stipend for each focus group they attended to value their time and participation. FROs relied on their local and participant expertise to determine the stipend value, ranging from USD 50 to USD 75 per focus group.

We organized focus groups that included parents or caregivers of children, youth, or young adults with behavioral health needs (*n* = 36) across six states. We determined the scheduling of the two focus group sessions in collaboration with partner FROs to align with the most convenient meeting times for their respective families. We set these optimal times at the outset of the project. Partner FROs then communicated the scheduled dates and times to potential participants, distributed the necessary meeting links, and confirmed member attendance. While all participants initially agreed to attend both focus group sessions, there was some attrition between the first and second sessions. FROs extended invitations to new participants to maintain group sizes, filling any gaps in the second session, leading to a small number of participants only attending one session. 

The majority of the sample was female and identified as female, with an average age of late 40s. Racial and ethnic-minority parents made up nearly half of the sample. Over 60% of participants were from frontier or rural settings. More than 80% of participants reported being the legal or biological parent. On average, though some participants were very new to working with behavioral health providers, caregivers reported having almost eight years of experience in using children’s mental health services. Over half of participants reported experience with special education programs or community-based mental health care, and about a quarter reported experience with child welfare, juvenile justice, or residential psychiatric care.

### 2.2. Data Collection

We developed detailed protocols in collaboration with the FROs to direct the focus group discussion, considering communication styles and social norms to foster a clear and engaging environment that would encourage active participation and dialogue. Table 1 outlines guiding questions for each set of focus groups. The project team used the protocols to facilitate a semi-structured discussion aimed at uncovering the expectations and aspirations of caregivers regarding the outcomes for their children, themselves, and their families from using mental health services. We also encouraged participants to articulate their views on what constitutes a positive or successful encounter with behavioral health services. Facilitators provided a broad definition of “behavioral health service” to encompass any form of support—such as peer support, respite care, medication management, therapy, and case management—delivered by a range of providers, including therapists, parent peer support providers, paraprofessionals, and care coordinators, across various settings, like private practices, community mental health centers, schools, hospitals, and residential facilities.

We held the initial series of focus groups in February and early March 2023, with a follow-up series in April 2023. We organized the focus groups to consist of four to six members of caregivers from the same community or state, with separate sessions for each group. We facilitated 12 focus groups: six groups in round one and six groups in round two. A research team member, accompanied by an FRO staff member or trained volunteer to ensure peer support and foster engagement, facilitated each focus group. The FREDLA Project Director or research assistant provided administrative support for each focus group and managed logistical aspects, such as the chat function, visual aids, and notetaking, during the sessions.

We recorded all focus groups and created verbatim transcripts from these recordings. We analyzed the collected responses using a two-pass in vivo coding process using inductive content analysis [18], which directly extracted participant expressions to summarize for the second round of focus groups. We used MAXQDA 24 Pro Analytics software [19] to create code clouds for visual guidance in Round 2 discussions. These clouds emerged from participants’ responses to the final question in Round 1, highlighting the three most important outcomes. The frequency of each mentioned word or phrase determined its font size in the cloud, visually emphasizing the most recurrent themes. 

The facilitation team introduced code clouds during the second round of focus groups and provided thorough explanations to each group. They addressed any clarifying questions and then introduced discussion prompts: (1) Is there something you disagree with here? (2) Is there anything that we talked about that isn’t reported here? and (3) Pick your top three most important outcomes from this code cloud. The discussions continued with participants to clarify the meanings of phrases in the word cloud, to assess the appropriateness of word or phrasing choices, and to differentiate the meanings of terms and phrases. These conversations provided additional data about participant preferences and priorities and were instrumental in refining the coding scheme and guiding the application of codes in future analyses. 

### 2.3. Data Analysis

Our analysis began with two rounds of inductive coding on the first focus group transcripts. This approach allowed us to identify the mental health outcomes that mattered most to parents/caregivers. We intentionally avoided a priori coding schemes to ensure the capture of fresh insights as participant perspectives emerged. The initial coding round involved two in vivo techniques, capturing direct quotes that precisely reflected participants’ views. We then refined, merged, and restructured these codes in a subsequent round. We coded the second set of focus group transcripts using the coding scheme from Round 1 transcripts but remained open to introducing new inductive codes as necessary. A second refinement and collapsing of codes followed, leading to the pooling themes from both rounds, centered around the top-three outcomes question. This process yielded a preliminary summary of responses to the mental health outcomes question for caregivers. 

Next, we presented this draft summary in two interpretive sessions with our partner FROs to refine terminology and gather feedback on the format and substance of the outcome lists. Informed by these discussions, we developed a comprehensive coding scheme. We then applied this refined scheme across all transcripts, with a final round of coding that included automated coding techniques and code merging. Two coders completed these analyses independently, achieving high interrater reliability (*κ* = 0.82). We resolved discrepancies between codes through collaborative discussion with the research team, ensuring a consensus-driven approach to final coding.

We generated code relationship matrices with code counts, focusing specifically on responses to the “Top Three” question in both rounds. This step was essential to determine the “Highest Priority Outcomes”. We calculated final code counts by averaging code tallies from each coder. Then, we identified outcomes with the ten highest code counts, particularly noting the outcomes prioritized by both groups. We repeated this analytic process for responses to all focus group questions and prompts within transcripts to broaden our understanding of other important outcomes reported by caregivers. This process allowed us to generate a list of “Additional Outcomes” that, while not ranked in the top three, were still considered significant by participants. 

## 3. Results

Table 2 details the highest-priority behavioral health service outcomes reported by caregivers of children, youth, and young adults with mental health needs. This table lists outcomes in descending order of the frequency reported by participants in response to the “Top Three” question over both focus group rounds. The entire list of mental health services outcomes reported by caregivers throughout the study is reported with example quotes in Table S1. 

In the text below, we describe the highest-priority outcomes identified by caregivers. Since many outcomes reported related to service system quality, these are grouped under one heading before reporting and describing other outcomes not related to service quality. For the group of service quality outcomes as a whole, and for each outcome unrelated to service quality, we also provide a summary of the values and perspectives underlying caregivers’ priorities. In summaries of participant values and perspectives—though example quotes for each outcome description are not provided within the text (they can be observed in Table S2)—illustrative quotes are provided within the text in these sections to support researcher interpretation.

## 4. Top Outcomes

### 4.1. Service System Quality Outcomes

Most caregiver responses centered on aspects of the service delivery process, such as accessibility, provider collaboration, and communication—elements typically categorized by researchers as system outcomes. These are distinct from the clinical or functional outcomes traditionally targeted in behavioral health care, like symptom reduction or school attendance improvement. Notably, this emphasis on service quality emerged despite only one of the four guiding questions explicitly asking about service delivery. Among the top outcomes, only three—knowledge and resources for mental health support, personal fulfillment, and improved functioning—did not directly relate to service quality. Additionally, two outcomes, effective communication and less judgment and stigma, encompass provider behavior and service quality. Priority outcomes reported by caregivers that were explicitly about service quality include (1) accessible services, (2) provider collaboration, (3) consistent and continuous care, (4) individualized care, (5) feeling supported and encouraged, (6) feeling respected, (7) well-trained providers, (8) cultural responsiveness, and (9) accountable service systems. Below, we describe each outcome’s meaning and explore why participants so highly prioritize service quality outcomes.

Caregivers universally identified access to necessary services and support as the most critical outcome for their families. In essence, caregivers called for a spectrum of community-based or in-home services and support delivered consistently, continuously, and adapting to the changing needs of families throughout their children’s developmental stages. Parents noticed a concerning decline in available services as children mature. Caregivers pointed out that these services often dwindle precisely at a developmental stage when older youth and their families need them most as youth are transitioning to young adulthood. Parent–peer support emerged as another vital need, reflecting a broad desire among caregivers for relatable and empathetic support systems.

Moreover, the narrative surrounding mental health care in schools revealed a significant shortfall, with caregivers wishing for more integrated behavioral health services. The urgency for respite care was also apparent, with parents lamenting the extreme measures required to receive necessary breaks from caregiving duties, including stays in the juvenile justice system. Caregivers also expressed frustration over well-documented treatment plans that failed to materialize into actual services and support. The struggles were amplified in rural communities, where specialists are scarce, and travel becomes an additional burden. Lastly, caregivers highlighted the flexibility of service delivery as a cornerstone of accessibility, especially in rural areas. Parents called for various service options, such as telehealth, weekend appointments, and in-home services, to accommodate working families.

Caregivers expressed that effective collaboration among all entities was also a critical outcome of mental health services. This collaboration extended beyond the immediate healthcare providers to include educators and family members, ensuring all parties were aligned in the child’s care plan. Furthermore, caregivers emphasized the need for all professionals engaged with their child to have a cohesive understanding of their child’s mental health needs and to work towards shared objectives. Caregivers specifically referenced a desire for family-driven care, which integrates the insights, preferences, and priorities of families and children themselves in developing and implementing effective treatment plans that are responsive to each child’s unique needs.

Caregivers reported prioritizing a consistent care trajectory that does not leave their children vulnerable at a time of mental health need or during critical transitions. Caregivers emphasized the need for a seamless transition from children and youth to adult mental health services. The continuity of care was particularly crucial when moving from more to less restrictive care settings. The struggle to secure an effective aftercare treatment plan was a common problem. Caregivers also expressed the desire for less frequent changes in the providers working with their child. They described how constantly changing providers interrupted the treatment process, slowing it down and inhibiting progress for their child.

Caregivers stressed the importance of tailored and holistic care approaches that acknowledge each child and family’s unique needs and circumstances. The core of this discussion centered around recognizing the child and family’s full narrative and culture. The need for flexibility and creativity from providers was another strong theme. Caregivers voiced concerns about an over-reliance on medication and a standard “one size fits all” approach, which fails to capture the unique complexities of their situations. Linking individualized care to youth- and family-driven care, caregivers pointed out that truly personalized treatment inherently requires providers to listen to and collaborate with family, as each family’s individuality directly informs the care plan.

Caregivers voiced a deep need for providers to offer affirmative feedback and positive reinforcement. Communication of unconditional support is seen as crucial for children’s self-esteem and future orientation. Caregivers also sought acknowledgment and validation from providers for themselves. Furthermore, caregivers reported the need for their children to feel that their concerns and aspirations are recognized and taken seriously. Caregiver narratives conveyed the desire for a support style that assisted them with the practicalities of care while providing the emotional backing and affirmation needed to navigate the complexities of raising a child with behavioral health needs.

Feeling respected was a central concern for caregivers engaging in behavioral health services. They expressed a need to be treated with dignity and respect by providers across various systems of care, including educational settings, and they associated this respect with professionalism. Caregivers understood respect as recognizing a parent’s expertise and engaging in dialogue as equals and partners.

Caregivers underscored the importance of expertise and professionalism in their children’s care. There was a clear call for providers to be adequately trained and compensated, recognizing their complex responsibilities. Caregivers voiced concerns about providers’ ability to be resourceful and proactive in connecting families with community support systems. Additionally, caregivers wanted providers to utilize intervention approaches and techniques supported by practice or research evidence and possess a strong understanding of the specific therapies they are implementing. The need for trauma-informed care was also frequently noted, with caregivers hoping for providers who are sensitive to and skilled in addressing past traumas and well trained and well versed in the nuances of trauma-informed care. Caregivers deemed this comprehensive approach to professionalism essential for fostering healing and growth, extending its importance to interactions with educators and first responders, often on the front lines of ensuring a child’s well-being.

The imperative for cultural responsiveness emerged as a pivotal outcome for caregivers seeking behavioral health services. They stressed the importance of providers who recognize and respect the cultural contexts and values that shape their family’s experiences. Acknowledgment of cultural context was reported as critical in understanding participants’ diverse racialized and ethnic backgrounds and navigating their varied family structures, such as those involving co-parenting arrangements or grandparent-led households.

Caregivers also reported a desire for accountability within service systems, calling for a higher standard of responsibility from providers and the overall behavioral health system. They voiced frustrations with a lack of accountability when issues arose, often feeling that blame was unfairly placed on them. Caregivers advocated for systems with clear ownership of actions, especially concerning the delivery of services outlined in treatment plans, the assignment of qualified providers, and the management of care that leads to improving or worsening a child’s condition.

*Why Service Quality Outcomes Matter:* Caregivers emphasized that service system quality is crucial for effective treatments, believing that clinical and functional improvements are hindered without key service qualities in place. One caregiver summed it up by sharing her son’s frustration: “Mom, if everybody would actually work together…it’ll help me function better”. Additionally, the stressors related to accessing care, like long commutes to treatment locations, can be counterproductive, exacerbating stress for the child and family. Ineffective service system operations, such as high provider turnover, can retraumatize children, as one caregiver noted: “My daughter doesn’t want to keep telling her story…over and over again”. Caregivers also linked their stress to concerns about their child’s future and the future absence of necessary supports for them. Caregivers saw strong provider rapport, achieved through consistent care, as vital for positive child outcomes, with one parent stating, “The effectiveness of your treatment team…boils down to the way your child feels safe with them”.

Further, participants often viewed mental or behavioral health issues as chronic, anticipating the need for long-term management rather than expecting significant improvement. One parent expressed the ongoing nature of the challenges: “This is a lifelong situation…”. This sentiment suggests that caregivers are not always hopeful for complete symptom remission but seek sustainable support, especially as their children transition to adulthood. The desire for a secure future for their children was a common theme, with a caregiver expressing, “…it would be very comforting to me that I would know that my kids would know how to take care of themselves or have guardianship…that they would be safe and live their best possible life”.

### 4.2. Individual and Family Outcomes: Knowledge, Resources, and Tools

Caregivers conveyed the crucial need for a comprehensive understanding and practical strategies to support their child’s mental health. They sought to gain skills to aid their children in managing emotional and behavioral challenges and to understand the underlying causes of their child’s anxiety, depression, or emotional dysregulation. Moreover, caregivers wished for providers to be well informed about individual mental health strategies and the broader landscape of community resources that could support their children and their families—and to share those resources with the family.

*Why it Matters:* A caregiver aptly summed up the value of such knowledge: “A better understanding of why behaviors or issues are happening and how to address what is triggering the behavior instead of viewing the behavior itself as a negative… it will help us be a better parent to them and to change maybe our ways of holding things together”. Other parents wanted to be able to support their child’s growth and healing by supporting their skill development at home and helping them practice and apply the skills and insights they are gaining from mental health services. “I know for me, the best way to support my daughter was really learning new and different skills that work better for how my daughter processes”. For other parents, these tools can be critical for coordinating and collaborating across the whole care team, and the parent is part of the care team. “You know… the parents are using kind of the same tools as the therapist and the educators, and yeah, shared tools”. The link between knowledge and access to care was significant, as caregivers reported that having the correct information was essential to accessing appropriate care. “When I ask questions, the people on the team should be knowledgeable about the resources in their communities”.

### 4.3. Individual and Family Outcomes: Effective Communication

Caregivers highlighted the need for clear and effective communication with professionals involved in their children’s care. They stressed the importance of regular and meaningful exchanges with providers, such as teachers, to discuss treatment plans, behavioral progress, and educational achievements. Caregivers also expressed the need for open lines of communication within the family unit, especially with their children. They aspired for an environment where they could openly share difficulties and struggles, aiming to support their child through challenges. The need for effective communication was also connected with access to care and collaboration among providers, illustrating that effective communication was also seen as essential to an interconnected support system where information flows freely and forms the basis of a collaborative, family-centered care approach.

*Why it Matters:* For many parents, effective communication skills were perceived as a vital facet of improved functioning and essential to independent living for their child. Caregivers believed that their child’s mental health condition could be better managed if their child learned to talk about their needs and concerns with them. “Just so they can open up to me… that’s all I’m just basically looking for”. Caregivers implied that much of their child’s reluctance to communicate honestly was due to social stigma: “… just my child being able to communicate more without fear, without the fear of being judged”. They also expressed the view that open communication would help reduce family conflict and mental health stigma: “I think you would see our family talk about the issue more… And I think that reduces stigma every single time. And that’s also kind of like how families get more united. They’re not afraid to talk about issues that they’ve confronted successfully”.

### 4.4. Less Judgment and Stigma

Caregivers expressed a desire for a societal shift in attitudes toward mental health. For respondents, this outcome closely intersected with access to care, indicating that perceived stigma often obstructed service access and affected community integration. Caregivers sought respect and understanding from providers, educators, family, and the broader community rather than blame for their children’s emotional and behavioral struggles. The stigma extended to uninvited advice on discipline from peers and family members, often inappropriately harsh and misguided, reflecting a lack of understanding about children’s mental health conditions. One caregiver painfully recounted, “I had somebody tell me that they thought I was too easy on my child, and I should beat my child and discipline them when he doesn’t behave”. These experiences illuminate the critical need for increased awareness and empathy from the community, which caregivers identified as essential to improving mental health outcomes for their families. 

*Why it Matters:* For parents and caregivers, the reduction in societal stigma was tied to improved functioning for themselves and/or their children because social norms and structures would change to accommodate their differences. As one parent stated, “I feel like …the thing that’s most important to him is a sense of belonging…which is like having a community, a group that you feel like ‘these are my people’. I belong here...They notice I’m here, and I’m here because I want to be here. And they want me too”. Another parent stated, “… I want him to be able to decide, ‘Oh, I really hated that. I’m not doing that again’ versus like, ‘you can’t try to do that. It’s not available”. Additionally, respondents felt that reduced stigma would help their child navigate the world more successfully as an adult. “If they say… I have to take some time off because I have a therapy appointment like, I think you should be able to be transparent to your employer or your teacher, whoever, and it should be acceptable if you need to have a mental health appointment”.

Further, caregivers believed that the lack of adequate support for their family from professional and informal systems stemmed from mental health stigma. Insufficient funding for mental health services—which impacted the ability of their family to benefit from mental health treatment—was a result of mental health stigma. “It really comes down to the stigma, and that’s why people are not wanting to fund the mental health system”. Additionally, caregivers’ social isolation—which contributes to the family’s struggles and need to rely on formal helping systems—was a result of the stigma surrounding their child’s mental health needs. “I’ve noticed that my circle has been extremely limited because there is a lot of judgment, you know, from other parents, especially parents who have not had to go through any of this”.

### 4.5. Personal Fulfillment

The aspiration for personal fulfillment emerged as a poignant theme among caregivers discussing desired outcomes of behavioral health services. They spoke of fulfillment, not just in the context of caregiving but also in their own lives, encompassing aspirations, such as pursuing personal and professional goals, having fulfilling social relationships, being capable of enjoyment and happiness, and enjoying life’s simpler roles. “To just be a mom sounds nice”, one caregiver mused, capturing the longing for the space to embrace and enjoy the fundamental aspects of parenting. Caregivers also voiced a yearning for the well-being and happiness of their entire family, dreaming of a life where each member, including their child, feels content and integrated into the community. Yet, the relentless demands of managing a child’s behavioral health often overshadowed personal and caregiver aspirations. 

*Why it Matters:* Caregiver narratives reflect a desire among caregivers for a balance that allows for self-care and personal growth alongside managing their children’s needs, which is essential for their sense of self and fulfillment. The examples shared by caregivers from their daily lives illustrated that most felt they simply did not have the option to focus on themselves, although some realized the importance of self-care. “There are so many times I’m canceling events, or I’m canceling things I want to do. Or I’m canceling doctor’s appointments because I have to make sure that they’re stable and they’re OK”. However, one caregiver described having a revelation that self-care would ultimately be essential to their ability to continue to be healthy and capable of supporting their child. “… because just being there for my kids isn’t enough because I was going to die, I was going to die sooner than later, and I wasn’t going to be there for my kids”. 

Another parent shared how daily stress impacted their ability to adequately support their child and attend to family relationships, which are also valued outcomes. “Like, I’m not present mentally, and those kinds of things can really just shut you down as a family because you can’t show up for each other in, like, the moments that you’re not in crisis”. Participants spoke about the fact that self-care and attending to their own needs would provide additional support for their child’s and their families’ well-being. “I want to be able to have time to focus on the needs of others that are also in my household, including myself, and that means rest and being able to take 5 min and just download…”. Additionally, caregivers investing in developing an adult peer social support network can support the whole family, particularly in times of need. “Being able to engage with my adult friends without feeling like a Mama on a nest sitting on these eggs, waiting for them to hatch. Because you get so isolated, and it’s horrific because when you need someone the most, parents get so intensely isolated because we’re not living”.

### 4.6. Improved Functioning

Caregivers expressed aspiration for their children’s enhanced well-being across various life domains. They envisioned functional improvement aligned with typical developmental progress, effective use of learned skills across a range of settings, better academic performance, and clear progress in treatment. One caregiver succinctly captured this hope for “normalization” within the context of adolescent life as “within range of what would be normal for a teenager”. Another parent stressed the importance of practical application of therapy gains, “employing new skills that they’ve learned” and ensuring their child uses these skills appropriately in social settings. Caregivers’ narratives revealed a comprehensive definition of improved functioning that extends well beyond clinical settings into school and potential employment, making milestones such as school attendance and job attainment indicators of success. These insights highlight a common thread among caregivers—a wish for their children to thrive, not just cope, which is central to their vision of effective behavioral health services.

*Why it Matters:* Many parents’ desire to see improved functioning in their child is tied to their worry for their child’s safety and well-being when the parent is not there. Caregivers want their children to be able to take care of and advocate for themselves, ensuring that their children will be protected in their absence:

“Because there’s no way that, like, someone is going to be as patient as I am with my children. And there’s no way that anybody’s going to work harder than me to make sure they have what they have. So, when they get jobs and things like that, and they’re not happy with some decision, it is so important for them to be able to speak up and say, ‘Listen. I’m not going to do anymore overtime. I cannot stay late today. I’m not going to do those things’ and to be able to do it respectfully is the hard thing”.

## 5. Discussion

This study is among the first to report the mental health outcomes that matter most to a diverse sample of families who utilize children’s mental health services, as disclosed directly by families. Although only one of the four questions presented in the focus groups was about the service experience, the most highly valued behavioral health service outcomes reported by caregivers in this study were about the service experience. Nine out of fourteen highly prioritized outcomes reported by parents and caregivers reflected characteristics of the service process: having accessible services that meet their needs; having providers that collaborate effectively with parents and other service systems; experiencing consistent, continuous, and individualized mental health care for their child; having well-trained, respectful, culturally competent, supportive providers; and accountable service systems. Two top outcomes—effective communication and less judgment and stigma—were not exclusively about the service systems but did apply to caregivers’ desires for the quality of mental health services and their desires for their child and family. Only three reported top outcomes did not encompass service systems in any way: improved functioning; personal fulfillment; and increased knowledge, resources, and tools to support their child’s mental health needs. 

Caregivers in this study hoped for many outcomes documented in other research examining parents’ or caregivers’ service goals and their formulation of treatment problems. These include a desire for generally improved emotional and psychological well-being [15], child safety [10], increased child and youth functioning, increased autonomy and independence, and improvements in specific behavior problems [10,13]. However, the findings in this study suggest that parents and caregivers place significant emphasis on system functioning and service quality outcomes. This emphasis is consistent with one study that found caregivers more critical of providers, services, and programs [9] but differs from another finding that caregivers prioritized improvements in symptoms and functioning significantly more than service quality or system outcomes [11]. The timing of data collection in this study, in early 2023, may influence the emphasis placed by caregivers on service quality and system outcomes; in the wake of the COVID-19 pandemic of 2020–2022, public behavioral health service systems faced unprecedented provider shortages [20,21] and caregivers may be reflecting on recent systemic problems directly stemming from or exacerbated by pandemic conditions.

### Implications for System of Care Practice and Research

The themes that emerged as priority outcomes for caregivers in this project reflect ongoing concerns and reported problems in the children’s mental health system, cited over forty years ago in the pivotal report from the Children’s Defense Fund, “Unclaimed Children: The Failure of Public Responsibility to Children and Adolescents in Need of Mental Health Services” [22]. In response to this report, the federal government established federal funding to support the development and expansion of systems of care across the United States [23]. This funding continues to support these efforts today, but caregiver reports in this study suggest that these systems remain fragmented, inaccessible, uncollaborative, understaffed, and undertrained.

For providers and mental health organization leadership, prioritizing these concerns of caregivers may be critical to keeping children and youth in mental health services; caregivers must consent to care, arrange payment, and often provide transport or arrange care. Thus, these project findings point to the importance of renewing organizational efforts to understand how to build and sustain mental health systems of care that deliver accessible, coordinated, and consistent care to children with mental health needs and their families—care that results in families having increased knowledge, resources, and tools for supporting the child’s or youth’s mental health needs, more effective communication skills, and reduced stigma from communities and service systems. To achieve this, behavioral health providers undoubtedly need more financial and staffing resources to support the additional time required for adequate and rigorous provider training, inter-organizational communication and service coordination, and sufficient managerial and supervisory support [24,25,26]. While policymakers place significant emphasis on service quality and system outcomes [27,28], providers report that funding to support the delivery of high-quality care continues to be insufficient [29]. Increases in resources could come through policies that authorize payment for care coordination activities or higher payment rates for empirically supported interventions requiring rigorous training [30]. Additionally, research attention to service quality and system outcomes, as of 2011, continued to lag behind empirical examinations of interventions targeting clinical or functional outcomes [14,31]. An updated review of research, examining studies from 2012 to today, is needed to understand if this trend continues.

Further, caregivers’ narratives often included themes about trauma and safety for their children and their families. Caregivers raised concerns about the lack of trauma-informed services and how service system failures and inconsistencies contributed to their child’s ongoing struggles with trauma. Caregivers also indicated that concerns about the safety of their child and their family contributed to their own trauma and related stress. Both observations suggest that system and organizational investments in trauma-informed practices and system structures, as well as in practices that keep children and families safe in times of crisis, may be critical to improving clinical and functional mental health outcomes for children and their families. Promising recent public investments and policies support the development of these systems through legislation encouraging the implementation of trauma-informed practices [32] and the recent rollout of the 988 mental health crisis hotline [33]. However, child mental health system research is needed to understand the impact of these initiatives on the trauma and safety experiences of youth and families who rely on behavioral health systems.

*Stigma in Child Mental Health:* Though significant research has focused on understanding the role of mental health stigma in mental health care access and its impacts on children and youth with mental health needs, as well as their families [34,35,36], public commitment to reducing this stigma is relatively limited. Recent research demonstrates the persistence of widely held beliefs by individuals and policymakers that parents and caregivers are the cause of their child’s behavioral health problems [37,38]. This belief may be due to limited investment in reducing this stigma and understanding the caregiver experience. Though public mental health administrators acknowledge the need for efforts to reduce stigma [39] and evidence suggests growing stigma around mental illness [40], compared to public expenditures and activities for behavioral health services and interventions, public activities to reduce mental health stigma are dwarfed. In the last ten years, less than half of all states reported coordinated efforts or initiatives aimed at reducing mental health discrimination [41]. Renewed attention to public health approaches to reducing mental health stigma—among providers, within organizations, and across communities—and assessing their effectiveness is needed [42].

*Caregivers’ Personal Fulfillment:* Caregivers reported that personal fulfillment—such as focusing on personal or career goals, having meaningful friendships, or having a sense of self-efficacy as a parent—was a notable finding not reported in other assessments of youth and caregiver outcome or service priorities for behavioral health care. Though caregiver employment and financial burdens are well documented for parents of children with emotional or behavioral disorders [43,44], much less research has examined the impacts of mental health services or interventions on caregivers’ personal well-being or fulfillment [14,31]. Given the association of parental mental health with child and youth mental health [45,46], the health, mental health, and well-being of parents or caregivers deserve the attention of mental health providers. This is an essential area for future behavioral health intervention and services research.

*Relationships Among and Between Outcomes:* These findings and reflections point to how the outcomes caregivers prioritize interact with each other, within and across outcome domains. Study participants may have reported some outcomes as vital because they intuitively understood how they would influence other outcomes they valued. Outcomes within the same domains may be interrelated: improved communication skills within a family may yield greater mutual understanding and enhance family relationships. Across outcome domains, caregivers indicate that service quality impacts the clinical and functional outcomes they hope for when accessing services. For example, participants indicated that less provider turnover and more trauma-informed approaches to care would help children achieve their service goals more efficiently. Clinical and functional outcomes, however, may also lead to other outcomes they value. For example, a reduction in clinical symptoms—especially concerning depression or other mood disorders—will contribute to increased safety and reduced stress for families. Increased safety would result in system outcomes, including fewer hospitalizations or other out-of-home placements. Additionally, parents and caregivers want support and encouragement from their community and providers and more support with care coordination because they feel it would lower their stress.

Attention to the pathways through which high-priority outcomes are achieved via accomplishing other outcomes may be an important feature of future research. Additional partnership with youth and families is needed to better understand their perceptions of these pathways, and intervention studies are needed to test their hypotheses about mechanisms through which the most desired outcomes are achieved. For example, studies on the impact of care coordination on youth and families must examine the impact of the intervention on family stress but also attend to and measure if families perceived the intervention to be provided in the context of other important outcomes, such as support, encouragement, high-quality communication, and consistency.

In addition to gaining a greater understanding of how outcomes relate to one another, measures for outcomes reported here must be identified and evaluated for validity and accuracy in partnership with youth and caregivers. The results of these efforts must be disseminated to researchers, research funders, policymakers, and providers, and barriers to integrating these outcomes must be assessed and examined. Given the increasing use of performance-based payment models [47] and growing gaps between research, policy, and practice in child mental health services research [48], centering the priorities of youth and families using these services is essential to closing the research-to-practice gap and upgrading outcomes in children’s behavioral health care.

*Limitations:* The findings reported here should be considered in the context of a few study limitations. First, though geographic and demographic diversity was sought in the sampling method, the sample was primarily female, and participants’ socio-economic status was not collected. The priorities and perspectives of caregivers may differ based on caregiver gender, socio-economic status, and experiences of urban versus rural living, and these differences were not explored or reported here. Further, this study presents only the perspectives and values of caregivers; evidence suggests caregiver priorities differ from those of YYAs using behavioral health services [9,11,13]. While our full study asked both YYAs and caregivers about their outcome preferences, findings for YYAs and how they differ from those of caregivers are reported elsewhere [17]. Finally, though geographic diversity was sought in the study design, no caregivers from the Midwestern region of the U.S. were included in the study, and the experiences and priorities of families differ significantly across state lines [49,50,51,52]. Variations in public and private mental health care systems, which are shaped considerably by state policy [53,54], may influence the outcome priorities caregivers report. 

## 6. Conclusions

This study’s findings provide fundamental new knowledge of the mental health outcomes most valued by caregivers of children with a wide range of behavioral healthcare needs. Participant narratives’ focus on service quality suggests that caregivers prioritize an accessible, respectful, and supportive treatment environment, underpinned by well-trained and culturally responsive professionals. Results also point to caregivers’ desire for consistent and harmonized care strategies that not only involve seamless cross-sector provider collaboration and care transitions but also integrate the insights and preferences of families and children themselves. Caregivers want providers and care teams to consider the complexity of the child’s needs, as well as those of the whole family, to craft a customized care plan. Caregivers also seek a balance of accountability in services, where the children and service providers are both held accountable. 

As a result of participating in behavioral health services with these qualities, caregivers hope to gain increased knowledge, resources, and interpersonal tools and techniques to support the mental health needs of their children and help them improve their daily functioning. Caregivers also want to see their children and families gain more effective interpersonal and professional communication skills, experience less judgment and stigma related to the mental health needs of their children from providers and their communities, and achieve personal fulfillment for themselves and their children. Establishing general behavioral health service priorities for children and families provides a foundation for additional research, inter-professional dissemination, and multidisciplinary collaboration. These actions are needed to ensure the alignment of research aims, research funding, and service financing to prioritize the outcomes most important to families.

## Figures and Tables

**Table 1:** Focus group questions.

Round One
What results do you hope for when your child uses services?
What results do you hope for yourself when your child uses services?
What results do you hope for your family when your child uses services?
What makes a good experience in using services?
From our conversation today, what are the top three most important outcomes to you?
Round Two
Is there something here you disagree with?
Is there anything missing that we talked about that isn’t reported here?
Pick your top three most important outcomes from this code cloud.

**Table 2:** Outcomes from behavioral health services that matter to caregivers: ranked in the top three by participants.

Outcome	Rank
Accessible services	1
Provider collaboration (with parent and other providers/systems)	2
Knowledge, resources. and tools (to support child’s mental health needs)	3
Effective communication (parent, child, and service systems skills)	3
Consistent and continuous care	4
Less judgment and stigma	5
Individualized care	6
Personal fulfillment (for parent and child)	7
Feeling supported and encouraged	7
Feeling respected	8
Well-trained providers	8
Cultural responsiveness	9
Improved functioning (for child and family)	10
Accountability and responsibility	10

## Data Availability

The data presented in this study are available on request from the corresponding author. The data are not publicly available due to participant privacy.

## Supplementary Materials

The following supporting information can be downloaded at: https://www.mdpi.com/article/10.3390/ijerph21020172/s1. Table S1. Additional outcomes from behavioral health services that matter to caregivers. Table S2. Caregiver high-priority behavioral health services outcomes.
