# Supplementary material for: Behavioral Health Services Outcomes That Matter Most to Caregivers of Children, Youth, and Young Adults with Mental Health Needs
Source: Int J Environ Res Public Health. 2024 Feb 1;21(2):172. doi: 10.3390/ijerph21020172 (PMC10887955; doi:10.3390/ijerph21020172)
Supplement: Supplementary file 1 [file ijerph-21-00172-s001.zip › ijerph-2776636-supplementary.pdf]

International Journal of Environmental Research and Public Health

MDPI

Table S1. Caregiver High-Priority Behavioral Health Service Outcomes.

Rank	Outcome	Quote
1	Accessible services	• Sorry, I think that another thing that I've noticed, with everything that everybody else is saying, that when they're younger, the services, there's more of them. But as the children get older, the services start dropping off. And the resources that our community has for us are not there.<br>• Can I just add one more thing that was very valuable to me was peer services and that every family should have opportunity for a youth, peer for the child and a parent peer for the parent peer service.<br>• But also, like, in the school environment, especially in the charter schools, I wish they had more behavior mental health services.<br>• To get what they need, they shouldn't have to spend 30 days in juvie so our parents can have respite.<br>• We shouldn't have to live in our van for an entire year just to get diagnosed with autism.<br>• What I want to put across is that these plans are made that end up making the providers look good, but then the kids don't get the services that are in their plan.<br>• So, in rural communities, it's really hard to find those specialists, and I'm lucky that it's only an hour away.<br>• The services in my area are horrible so… I'm going to need to move to a different county probably because I just don't have—I don't have the services that I need to help my (kids).<br>• A good service will also be having, like, different types of services, especially in the rural community. Like telehealth, weekend appointments, because a lot of parents do work….like in-home services. That's what I prefer as well, but it's a hassle to get any type of services within home or just telehealth. You have to find specific providers that do that. So, it's hard when you work and can't find a lot of providers to offer that.

2	Provider collaboration (with parent and other providers/systems)	• But for us, having everybody at the table that we felt had a valid place at that table made a huge difference. The entire system of care that [child's name] was receiving with all of their school interventions, their behavioral interventions, their psychiatric interventions—everybody was on the same page. Everybody was looking at the same treatment plant. Everybody had the same goal.<br>• I mean, it's confusing because-- that's the other thing, nobody is ever on the same page. And there's too many, you know, like, chiefs in the tribe, it's, like, who am I supposed to listen to?<br>• Also, the parents need to have input, and not just be heard but be listened to; because I get a lot of that, 'We hear you. We hear what you're saying.' But are you listening?<br>• It should be based on what this child feels they need and then go from there with a plan. Because if we create a goal, and it's different from the child, they're not going to work nearly as much or nearly as hard to achieve those results as they would as if they feel like their plan was their goal oriented.
3	Knowledge, resources. and tools (to support child mental health needs)	• A better understanding of why behaviors or issues are happening and to address what is triggering the behavior instead of viewing the behavior itself as a negative…<br>• What's more, even if they can know without, you know, without anybody else knowing that they didn't tell us or their therapist or their counselor and told us, but it will help us being a better parent to them and to change maybe our ways of holding things together.<br>• Sometimes my kid just all of a sudden will erupt, and it came from nowhere. And that's—they don't teach you anything on how to control a child during that, because nobody really knows. They can teach you holds, they can teach you how to protect yourself. They can give you numbers of resources that you can call during the time, but a class is never going to teach you what you need for your child. And if you don't do these classes, they're like, oh, well, you're not a good parent.
3	Effective communication (parent, child, and service systems skills)	• I also value having a time each week or month to talk to the provider individually without child in room and really discuss the progress and struggles and work out a plan to tackle them as a team.<br>• That if she has difficulties or struggles, she'd be able to talk to us through them, and that we would be able to provide with her whatever support she needs…<br>• I would like for the family to be able to tell each other when something's wrong or bothering them without the explosion and triggering.

4	Consistent and continuous care	• I think my child benefits the most when we have someone who's not just like, rotating in and out, or when we have multiple providers over the week.<br>• I think there's a strong need for transitional services from child to adult…. Sometimes it's hard to get those. Some of them fall through the cracks. I have trouble right now, and it's been difficult because, like for me, I try to call and I don't get the right people, and they don't know who to talk to. And I don't know who to ask for, so I get kind of lost…<br>• …we begged and pleaded for him to not be released until they had a good treatment, aftercare treatment plan for him. I'm still right now, they released him without that… and I'm being told, 'Oh, I'm sorry we dropped the ball with the insurance. Oh, I'm sorry we didn't follow through with getting the referrals.'
5	Less judgment and stigma	• It's blame game when it's the parents. Oh, this is your fault for the way your child behaves and things like that.<br>• Were you on [drugs] when you're pregnant with them and that's why they're all messed up?' … But there's so much stigmatism [sic], and there's so many assumptions that you did something wrong and caused it with your child.<br>• One thing, if I could have a wish, I would just wish better understanding amongst people like I had said before, extended family members, but not only that, caregivers too. I had somebody tell me that they thought I was too easy on my child, and I should beat my child and discipline them when he doesn't behave….They actually told me that I should get a 2 by 4 and beat my child while they can all stand around and watch and they said it in front of my child.<br>• Non-judgmental providers
6	Individualized care	• Having opportunities to work with professionals with enough time for them to really think about the whole child, the whole family, and an integrated set of services…<br>• It's like we continue to do the same thing over and over and there has to be like this button that we reset and regroup and then go back at it a different way.<br>• I want them to look at him and start at him with a blank slate and create something new for him and learn from their experience with him so that they might be able to have some more information to their toolbox that might help them with the different kid. And I find that a lot of times, when you go to people, 'well, this is what we do for this situation.' Well, then it's not going to work because my situation is not your situation, and trust me, if you think that the outcome there's gonna be an outcome that is kind of like everybody else's outcome, my son will put you for a spin. He will show you there's all kinds of other things. He's got all kinds of stuff in his back pocket.<br>• And then I think individualized care, individualized treatment and family driven, because you can't individualize the treatment if you're not family driven, because every family is unique.

7	Personal fulfillment (for parent and child)	• To just be a mom sounds nice 😊<br>• That's always been my goal, is a happy, healthy child. I can't say for peace because a happy, healthy teenager is chaos… But it's a different kind of chaos. It's one that brings a smile to your heart instead of tears to your eyes.<br>• That's my biggest challenge, is using most of my processing speed and space, to always being propping up and all this stuff, and you know I don't have enough brain space for myself, let alone, you know, going out to the world and working. And that's that would be what it looks like would be to have the freedom, for me to do my goals, my life desires and, you know, do things beyond myself and my kid.<br>• I think for me it would be that I can go out and get a job or do a job and not have to worry, like the other parent said, about my kids—for my kid, being able to keep herself safe and keep her home safe.
7	Support and encouragement	• I would like to see more of them letting the children know no matter what amount of wrong you have done in the past, or now, that you still have a bright future.<br>• I want positive feedback…<br>• So, I feel like a result for me would be to have enough therapy and interventions and supports in place so that I don't feel the need to constantly micromanage everything about my children's care so that I can just enjoy being their mom.<br>• Having other parents have talked to like, a support group for parents as well to continue to have support group for parents as we support our children.<br>• That, just having that advocate for, that good support that's on my side…<br>• Basically, I really value them when they validate my feelings or my perceptions and thoughts before they, you know, move on in the conversation or before we address a solution.<br>• My thing is I need my child to feel validated that their concerns—their emotions, their desires, their goals for themselves—are valid and they're achievable.
8	Feeling respected	• And so those are good experiences, when the parent doesn't feel like they're being talked down to and is heard.<br>• I feel like the assumption is that I don't know what I'm talking about or that I'm not educated. So … I wrote that I'm treated as a human first, that my children are literally treated as humans, and that their health matters too.
8	Well-trained providers	• I think I'll kind of the gist of what they're saying is that it's not a professional. The jobs aren't professional, so the pay is poor. The turnover rate is high, and so the kids are the ones suffering because the perception is that this is a, you know, a quick minimum—just barely over minimum wage paying job that someone could take, but they are given a lot of

		responsibility with the kids' mental health. And, they're supposed to be the professionals with this? If you're copying pasting something that an 8-year-old can get off the Internet, how is that helpful?<br>• …sometimes that they prefer less challenging cases…<br>• I would like to see things, like, you know, the team saying something, and I say, OK, cool, great, let's get that done. Have them, you know, tell me what to do. Like getting my child involved with adult long-term care services. And, basically, it's, like, 'OK, we're on that' and it's like, 'OK, that's great.' And six months later, it's like they look at me like, 'So what have you done with that?' Like, sorry, you're the professionals. You know who to call. You have the, you know, the back room, you know, interactions, I'm assuming—or you at least have the keywords that you know will help my kid get on, you know, long term care.<br>• Like I've said with many, many [providers], when they say we're doing counseling with the kid, I say, what is the actual like counseling that's being done with the kid? Like, are they doing EMDR or CBT or whatever? What is the actual like counseling that's being done, and you get blank stares. 'No, we're just doing counseling. We're going to build rapport.'<br>• So, I would like the counselor to do things like, you know, not tell my kid, 'Well, that's in the past. You don't have to worry about that now. They don't do that at school anymore.' And it's like that doesn't help the trauma that she went through. You're not helping the trauma.<br>• Education of professionals. Because we shouldn't have a police officer telling you to beat your kid. We shouldn't have a hospital going, there's nothing wrong with your child. We shouldn't have a therapist going, oh, it's all the parent.
9	Cultural responsiveness	• I just feel like when it comes to mental health, you also have to consider the person's, the individual's culture as well. That's very, very important from top to bottom and you have to honor that.<br>• So, for me it was important, especially for my daughter. I don't know if she shared she has two homes, and I have her dad's not very receptive on getting therapy or even when she was having like different moments in her life that was causing her to self-harm. So, to me that was important for me for them to understand that, you know, like there's two different dynamics, family, as well and two different point of views as well.
10	Improved functioning (for child and family)	• …that we would just be within range of what would be normal for a teenager.<br>• employing new skills that they've learned…<br>• that they know to utilize them, and utilize them when appropriate, application of them when we are in public settings like a park or library…<br>• …just pull your F's up. I don't care if it's D.

		• I want to see growth. I want to see my child, you know, being the best child they can be.<br>• I'm a fan of seeing measurable progress and data based on their assessment charts.<br>• It worked out and so this child went from struggling with C's and D's, to an honor roll student. We had only one absence for the entire school year. The year before, they kept calling me so much she ended up with 18 absences that year.<br>• And then there's also the biggies, like, he would be employed.
10	Accountability and responsibility	• And you know, nothing's being done. It's hot potato-hot potato. It's always the parent's fault. It's never the provider's fault. And accountability isn't there.<br>• We expect the kids to be accountable. I want the adults accountable as well, for failures or lacking.

Source: Transcribed audio-recordings and chat notes from video conference.

Table S2. Additional Outcomes from Behavioral Health Services that Matter to Caregivers.

Rank	Outcome	Description	Quote
11	Being heard	Caregivers want providers to listen to families and their children about their needs and work for them—believing them and trusting their expertise. This is related to providers and schools not responding effectively to caregivers raising concerns about the needs of their child. It was particularly problematic when related to the child's safety.	• ….there's just nobody listening. We're screaming for help and we're not getting it because other people have more money or more resources.<br>• I called them the next day and I said, 'Are you hearing me? Now, do you hear now? She needs counseling.'<br>• It is hearing the concerns, hearing the whys it may not work and instead of pushing us, you know, you've got to do this kind of thing.
12	Peace and safety	Caregivers want to be confident that their child is safe, their family is safe, and there is calm and harmony in the home. There are no emergencies, and family or household conflict is within the normal range. This outcome is particularly important in reducing caregiver stress and worry.	• …to have the ability to keep herself safe and for my husband and I to also feel safe around her.<br>• The outcome I wanted was peace in my household. I wanted stabilization.<br>• I wrote down peace with within normal, you know, within the normal range with teenagers...<br>• For me, for my family, being whole in one household together, talking and laughing and having a good time, that's all I really want.

12	Less stress	Caregivers want less personal stress, which stems from coordinating all the care and advocating for their child's mental health needs, worry and fear for the safety and wellbeing of their child, and frequent conflict in the household. Caregivers report the perspective that their level of stress contributes to the family functional impairments and household stress.	• I mean, I honestly, I feel like with our situation it's just, for the whole family, I want us to feel less stressed.<br>• And then, like everybody says, less stress because it's stressful to be the like, the only advocate for your child as well.<br>• We are literally doing our best to prepare for the next nuclear fallout. So, like, not living in the constant anticipation of doom.<br>• I want to worry less… I worry that I'm going to walk in and I'm going to find her, um, not alive. I have been like, she's gonna have harmed herself.
13	Symptom reduction/ improved symptom management	Caregivers want to see their child's mental health symptoms reduced, especially in relation to depression and anxiety. They also want to see them reduce their impulsive behaviors, manage their emotional responses better, and learn how to live with their mental health needs.	• I think one of the simplest things is he would express being happy and not being anxious.<br>• My daughter would have less anxiety.<br>• Child is happier.<br>• Not suicidal, depressed or anxious<br>• …be accustomed to live with that anxiety.<br>• less meltdowns
14	Improved community integration	Caregivers want their children to be able to engage in community and school-based activities, even if support or accommodations are needed, and to derive enjoyment and a sense of belonging from those activities.	• More positive community interactions, being able to feel safe with sending my child to summer camp.<br>• Better able to do what he wants at school and in sports.<br>• Able to do day to day things all kids like to do and enjoy them
15	Increased understanding	Caregivers want others (providers, extended family, society) to understand their child and their family, but they also want family members to understand their child more as well. This outcome also refers to mutual understanding between caregivers, their child, and the rest of the family.	• I wanted the family, including myself, to learn to participate with each other more. And understanding or compromising.<br>• Harmony and understanding within the immediate family unit.<br>• There needs to be more understanding of the certain circumstances and what's going on.

16	Reduced system involvement	Caregivers reported measuring success for their child by how often they were out of the home for mental health care. Other caregivers reported desiring to keep their child out of involvement with the justice system through police contact or contact with child protective services, sometimes initiated by schools or other providers or required to access mental health care. Children may be out of the home due to their inability to be safe, but they may encounter trauma in their out-of-home service experiences.	• …. the threat hanging over your head that if you don't do it my way, or you don't do it this way, or you do it differently, well, that might, you know, trigger a DCS (Department of Child Services) call.<br>• So, my idea of a successful outcome would be that my daughter could get home and live with us, and we could be a thriving family.<br>• You know, using the number of times we are required to take this child to the doctor, the emergency room, and outpatient, and inpatient—whatever—use all of those, and seeing a decrease of those as a standard for how well these treatment plans are working.<br>• I feel like for my family, especially with my older daughter, when we are seeing success is when she is able to be home.<br>• As a parent, the most extreme success measure for us was the lack of hospitalization.<br>• Because when they have an episode or something where they might get by violent, the automatic response is to call the police.
17	More time from providers	Caregivers discussed the need for providers to spend more time with their child and their family in order to provide individualized care and that providers needed to offer services at times that worked for their families, in order for services to be more accessible. Also, caregivers asserted that providers needed to allow their child to stay in services longer because their mental health needs would not be fixed quickly or easily, or that some level of support may be needed always.	• Because is not, they're not taking the time to say, hey, this is different. Let's analyze. Let's see where this is coming from.<br>• I think giving them time to be able to, I mean, some of these things just aren't going to be addressed. It's not like a one like, quick thing.<br>• Like, some of our kids need to stay in the same place for five, six, ten years so that they can feel comfortable enough to move on<br>• I think they should spend more time with them and just getting their—asking questions. How are you doing today? Do you think the medicine is working? And then it's ready to write out another prescription?<br>• But the other thing about a good experience is how many people—how many providers that we've had are watching the clock.

18	Appropriate use of medication	Caregivers wanted effective and appropriate medication management for their child's mental health needs. While most caregivers were grateful for the support and help those medications provided, particularly for depression and anxiety, they often felt that they were prescribed too quickly and not re-evaluated often enough.	• So, one of the ways that we measured whether the treatment plan was working, whether what we were doing was being effective for her, was when we were able to start slowly reducing the frequency of medications, the number of medications.<br>• I feel like there needs to be a lot more frequent review of the amount of medication, and whether it's even necessary.<br>• They always want to find a plan to put them on medication when there's other things out there where you can help a child without being on medication.<br>• Medication is, I feel, is a way of the teachers and them not having to deal with your child.
19	Improved relationships	Caregivers hoped that their child would improve their interpersonal relationships, with their family members, their caregivers, their peers, and with other adults. They wanted their child to be able to have good health and functional and rewarding social and familial relationships.	• And what I want for her is to learn how to pick her friends….<br>• OK, I would like to see more of—I know siblings fight…. you know, that we'll all be able to get along, you know, love on one another.<br>• Less fighting, More giggling.<br>• That we can just enjoy being together without tension<br>• Be a healthy family unit.
20	Positive provider rapport	Caregivers wanted providers to show warmth, kindness, caring, and unconditional positive regard to their child and to themselves.	• When I feel like they understand and like my child for the wonderful person he is and both of us can tell.<br>• A provider who truly cares.<br>• I want a provider that cares about my child success and isn't just clocking in.<br>• For a provider to show empathy and understanding toward the parent and what they are going through.

21	Increased ability to trust	Caregivers reported wanting to increase their trust in the service system and their providers, as well as increase their ability to trust their own child.	• ...trust that they are capable of handling their emotions and handling their situation …<br>• it's hard to trust after you've been burned by providers.<br>• Gaining trust for my oldest teenager and being able to believe what she is telling me.<br>• Being able to receive the services with SAFETY and Confidentiality.<br>• I just want to say, just make sure that the person you're dealing with... make sure that you can trust them.
22	Motivation/structure for change	Caregivers wanted providers that could actively provide structure for behavioral health improvement as well as motivation for the child to engage in treatment and achieve their treatment goals.	• I would like to see more of them letting the children know no matter what …that you still have a bright future—that it's not too late for you to stop what you're doing and to turn your life around to get to where you want to go, and the things that you want to be, that they write down their goals.

Source: Transcribed audio-recordings and chat notes from video conference.
